# Evaluation of a research diagnostic algorithm for DSM-5 neurocognitive disorders in a population-based cohort of older adults

**DOI:** 10.1186/s13195-017-0246-x

**Published:** 2017-03-04

**Authors:** Ranmalee Eramudugolla, Moyra E. Mortby, Perminder Sachdev, Chantal Meslin, Rajeev Kumar, Kaarin J. Anstey

**Affiliations:** 1 Centre for Research on Ageing, Health and Wellbeing, Research School of Population Health, College of Medicine, Biology and Environment, Australian National University, 54 Mills Road, ACT 0200 Canberra, Australia; 2 Neuropsychiatric Institute, Prince of Wales Hospital, and Centre for Healthy Brain Ageing (CHeBA), School of Psychiatry, University of New South Wales, Sydney, Australia; 3 Academic Unit of Psychiatry and Addiction Medicine, College of Medicine, Biology and Environment, Australian National University, Canberra, Australia

**Keywords:** Neurocognitive disorders, dementia, Mild cognitive impairment, Diagnostic algorithm, Longitudinal, Cognitive aging, DSM-5

## Abstract

**Background:**

There is little information on the application and impact of revised criteria for diagnosing dementia and mild cognitive impairment (MCI), now termed major and mild neurocognitive disorders (NCDs) in the DSM-5. We evaluate a psychometric algorithm for diagnosing DSM-5 NCDs in a community-dwelling sample, and characterize the neuropsychological and functional profile of expert-diagnosed DSM-5 NCDs relative to DSM-IV dementia and International Working Group criteria for MCI.

**Methods:**

A population-based sample of 1644 adults aged 72–78 years was assessed. Algorithmic diagnostic criteria used detailed neuropsychological data, medical history, longitudinal cognitive performance, and informant interview. Those meeting all criteria for at least one diagnosis had data reviewed by a neurologist (expert diagnosis) who achieved consensus with a psychiatrist for complex cases.

**Results:**

The algorithm accurately classified DSM-5 major NCD (area under the curve (AUC) = 0.95, 95% confidence interval (CI) 0.92–0.97), DSM-IV dementia (AUC = 0.91, 95% CI 0.85–0.97), DSM-5 mild NCD (AUC = 0.75, 95% CI 0.70–0.80), and MCI (AUC = 0.76, 95% CI 0.72–0.81) when compared to expert diagnosis. Expert diagnosis of dementia using DSM-5 criteria overlapped with 90% of DSM-IV dementia cases, but resulted in a 127% increase in diagnosis relative to DSM-IV. Additional cases had less severe memory, language impairment, and instrumental activities of daily living (IADL) impairments compared to cases meeting DSM-IV criteria for dementia. DSM-5 mild NCD overlapped with 83% of MCI cases and resulted in a 19% increase in diagnosis. These additional cases had a subtly different neurocognitive profile to MCI cases, including poorer social cognition.

**Conclusion:**

DSM-5 NCD criteria can be operationalized in a psychometric algorithm in a population setting. Expert diagnosis using DSM-5 NCD criteria captured most cases with DSM-IV dementia and MCI in our sample, but included many additional cases suggesting that DSM-5 criteria are broader in their categorization.

**Electronic supplementary material:**

The online version of this article (doi:10.1186/s13195-017-0246-x) contains supplementary material, which is available to authorized users.

## Background

Revised criteria for diagnosing dementia and mild cognitive impairment (MCI), now termed major and mild neurocognitive disorders (NCDs), respectively, in the Diagnostic and Statistical Manual of Mental Disorders, Fifth Edition (DSM-5) [1], has the potential to significantly impact on clinical and research settings. Recent reviews [2, 3] note the increased clarity and structure in DSM-5 NCD for assessing cognitive impairment, decline, and functional impact when compared to DSM-IV dementia or International Working Group (IWG) criteria for MCI [4]. The clearer criteria and greater emphasis on objective measures mean that the DSM-5 NCD categories should be easier to operationalize in large-scale studies of ageing using a psychometric algorithm. Algorithmic approaches to diagnosing NCDs are particularly valuable in resource-intensive population studies [5] and in settings where there is limited access to biomarkers and clinical services. Globally, most dementia cases occur in such settings [6]. Algorithmic approaches to DSM-IV and DSM-III-R dementia diagnosis have been previously published with agreement ranging from κ (Cohen’s kappa) = 0.63 to 0.84 [5, 7, 8]. No study has as yet examined the algorithmic diagnosis of DSM-5 NCD. The present study fills this gap.

Given that both major and mild categories of NCD are designed to be age- and etiology-independent syndromes, it is expected that, when applied to older adults, the prevalence estimates would be higher than for the more ‘Alzheimer’s-centric’ DSM-IV dementia category [2, 9], whereas MCI criteria [4, 10] are much broader and are not age- or Alzheimer’s disease (AD)-specific. Field trials of DSM-5 suggested a similar prevalence of DSM-IV dementia and DSM-5 major NCD [11]. However, a number of recent studies [12–14] report differences between the DSM-5 and existing diagnostic systems, with one reporting increased prevalence of diagnosis with DSM-5 criteria relative to DSM-IV and MCI [14], and others reporting decreased diagnosis relative to systems such as 10/66 criteria [12], Petersen MCI criteria [13], and IWG-MCI criteria [14, 15]. The variance in findings may reflect differences in the diagnostic systems used for comparison, sensitivity of different cognitive batteries, as well as the samples studied (e.g., memory clinic [14], population-based cohort [12, 13, 15], middle-income nations [12, 14]). In the context of these mixed findings, it is important to better understand the implications of applying DSM-5 NCD criteria to existing epidemiological studies with well characterized samples that have been followed longitudinally with neurocognitive diagnoses.

The aims of the present study were twofold. The first aim was methodological and sought to develop and evaluate a psychometric algorithm to assess participant data against criteria for the following diagnoses: DSM-5 major NCD, DSM-5 mild NCD, DSM-IV dementia, and IWG MCI. Algorithmic classification was compared to diagnosis of the same categories by experienced clinicians (expert diagnosis). The second aim was to examine the overlap between expertly diagnosed DSM-5 NCDs, DSM-IV dementia, and MCI, and characterize the groups in terms of their neuropsychological and functional profiles.

## Methods

### Participants

The participants were from the Personality and Total Health Through Life Project (PATH) which has been previously described [16]. Briefly, we recruited participants who were residents of the city of Canberra and adjacent town of Queanbeyan, Australia. Participants aged within three narrow cohorts (20–24, 40–44, and 60–64 years) were sampled randomly from the electoral roll and invited to participate in a study on the risk and protective factors for common mental disorders. Enrolment to vote is compulsory for all Australian citizens. The study protocol was approved by the Australian National University’s Human Research Ethics Committee (Protocols: 2009/039; 2009/308; 2012/074; 2006/0314; 2002/0189) and participants provided written informed consent after receiving a complete description of the study. A total of 7485 consented to participate. The present study focuses on the older age cohort whose sample size at wave 1 (data collection 2001–2002) was 2551 (58.3% of the cohort’s random sample). Participants were re-assessed every 4 years on a broad range of sociodemographic, health, lifestyle, and neuropsychological measures. Sample retention has been high at each wave (between 85.4% and 88.8%). This study reports data from the 12-year follow-up of the older cohort who were aged 72–78 at wave 4 (data collection 2014–2015).

### Interview and assessment

**Fig. 1:**
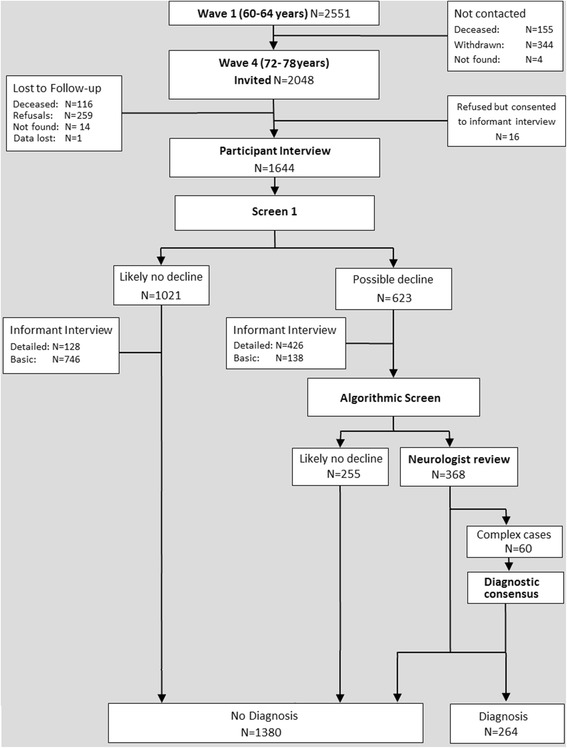
Flow of participants through the PATH study and through wave 4. Diagnosis refers to DSM-5 neurocognitive disorders, IWG MCI, and DSM-IV dementia

Of the 2048 participants contacted for follow-up at wave 4, 116 were deceased, 259 refused, and 14 were not found (Fig. 1). Data were obtained from individual face-to-face or telephone interviews conducted with 1644 participants by trained research personnel, including demographic, general health, anthropometric, physiological, and neurocognitive measures.

### Demographics, depression and general health survey

An interviewer-administered survey collected data on the level of education, psychological measures, substance and medication use, psychiatric and medical history, including recent major surgery, activities of daily living, housing, home or personal care, and non-English speaking background. Depressive symptoms were screened using the self-report screen for DSM-IV criteria for depression, the Patient Health Questionnaire (PHQ-9) [17].

### Cognitive assessment

A battery of neurocognitive measures was developed to address each of the domains described in the DSM-5 [1] (see Additional file 1: Table S1), and administered by trained research interviewers. Measures were selected on the basis of sensitivity to dementia and age-related cognitive impairment as well as efficiency of administration and scoring. Data on behavioral changes were obtained through the informant interview (see later). Briefly, the following measures were used to assess each of the domains: complex attention (Symbol Digits Modalities Test [18], Trail Making Test A [19], Reaction Time Test [20]); executive function (Digit Span Backwards [21], Trail Making Test B (19), Stroop Color Word Test [22], Zoo Map Test [23], Game of Dice Test [24]); learning and memory (California Verbal Learning Test [25], Benton Visual Retention Test (Administration B) [26]); language (Letter Fluency [19], Boston Naming Test-15 item [27], Spot The Word Test [28]); perceptual motor (Purdue Pegboard [29], Ideomotor Apraxia Test (IAT) [30], Benton Visual Retention Test (Administration C) [26]); social cognition (Reading the Mind in the Eyes [31]). Details on test measures are provided in a supplementary methods section (see Additional file 1). Scores were converted to *z* scores by normalizing relative to the whole wave 4 PATH sample data stratified by gender and education (low: 5–10 years, medium: 10–15 years; high: 15+ years).

### Screen 1

The data for the 1644 participants assessed at wave 4 were screened for signs of decline based on the criteria detailed in Additional file 1. Briefly, this included either a previous PATH diagnosis of dementia or a mild cognitive disorder, or evidence of current objective cognitive impairment (based on performance ≤6.7^th^ percentile on at least one cognitive measure, or Mini-Mental Status Examination (MMSE) ≤24), and evidence of subjective decline on the Memory and Cognition Questionnaire (MAC-Q) [32] or decline on the MMSE of >3 points since wave 3, or consistent MMSE ≤24 at waves 3 and 4. Of the participants meeting criteria for any of the above (*n* = 623), the majority (*n* = 426) had a detailed informant interview. Of the remaining 1021 participants not meeting the criteria, most (*n* = 746) received a basic informant interview (Fig. 1).

### Informant interview

Participants (*n* = 1438) consented to have an informant (spouse, friend, neighbor or relative) interviewed by telephone regarding the participant’s changes in cognition and activities of daily life. The basic informant interview comprised the Bayer instrumental activities of daily living (IADL) questionnaire [33] and the Informant Questionnaire of Cognitive Decline in the Elderly 16-item Short Version (IQCODE) [34]. The detailed informant interview comprised the Bayer IADL, IQCODE, Dysexecutive Questionnaire (DEX-Q) [23], and Neuropsychiatric Inventory (NPI) [35], as well as questions on medical history (Parkinson’s disease, Alzheimer’s disease, other dementia, stroke, psychiatric diagnoses, memory complaints), recent behavior including symptoms of delirium, psychosis, hallucinations, alertness and physical function, sensory or motor loss, and onset and progression of cognitive difficulties. The DEX-Q [23] collected data on executive difficulties affecting social and daily activity. The NPI [35] collected data on non-cognitive symptoms of MCI and dementia.

### Psychometric algorithm

Those identified by screen 1 (*n* = 623) had all interview and informant data entered into a case file spreadsheet. To minimize effects of non-response bias, case files with missing informant data (*n* = 59) were also screened by the algorithm. The algorithm combined the neurocognitive assessment data with the informant and survey data on medical history to operationalize criteria (criterion met/not met) for each diagnostic category: DSM-5 major NCD, mild NCD, DSM-IV dementia, and MCI (see Tables 1 and 2). Details of the neuropsychological battery are provided in Additional file 1. Cognitive scores were standardized relative to the gender- and education-stratified norms (from the whole PATH 60s sample at wave 4) and converted to *z* scores. Severe cognitive impairment was defined as a *z* score < –2.0. Given a lack of consensus in the literature regarding appropriate cut-offs for defining mild cognitive impairment, separate algorithmic categories were created using *z* score > –2.0 and ≤ –1.0, and > –2.0 and ≤ –1.5. In addition to the diagnostic categories of interest to the current study, the algorithm also classified participants according to other categories (e.g., age-associated memory impairment [36], age-associated cognitive decline [37], DSM-IV mild NCD, etc.). Participants not meeting criteria for any diagnostic category were classified as “normal”. Those meeting criteria for at least one diagnosis (*n* = 368) had their data reviewed by the research neurologist (Fig. 1).

**Table 1:** Operationalization of DSM-5 major NCD and DSMIV dementia within the algorithm

DSM-5 major neurocognitive disorder			DSM-IV dementia		
Criteria		Algorithm	Criteria		Algorithm
A. Both of the following:			A. Both of the following:		
A1	Concern of self or informant of significant cognitive decline in one or more cognitive domains	MAC-Q >24 *or* IQCODE >3.31 *or* recent doctor’s consultation about cognitive change *or* informant reported worsening of everyday cognitive function^a^	A1	Memory impairment	Mean *z* score for the learning and memory domain ≤ –2.0.
A2	Substantial impairment in cognitive performance in one or more cognitive domains	Mean *z* score for one or more of the following domains is ≤ –2.0: complex attention, executive function, learning and memory, language, perceptual/motor, social cognition *or* mean decline in performance between waves 3 and 4 that is </=-2.0 below norms on select tests^f^.	A2	Substantial impairment in cognitive performance in one or more cognitive domains	Mean *z* scores ≤ –2 for one or more of: executive function, language, praxis^b^, gnosis^b^ *or* mean decline in performance between waves 3 and 4 that is </=-2.0 below norms on select tests^f^.
B	The cognitive deficits interfere with independence in everyday activity	Any self-reported problems due to memory on HRS-IADL^c^, *or* need for household/personal care help, *or* Bayer IADL >3.12, *and* (if A2 social cognition impaired, then also informant-reported^d^ change in social behaviors or emotion recognition)	B	A1 and A2 each cause significant social/occupational dysfunction and represent a decline	Any HRS-IADL^c^ problems due to memory, or need for household/personal care help, *or* Bayer IADL >3.12, *and* (if A2 praxis or gnosis impaired then also informant-reported^e^ difficulties in everyday praxis and object recognition)
C	The cognitive deficits do not occur exclusively in the context of a delirium	Informant reports deficits started 6 months or more ago, *or* informant reported delirium signs present for less than duration of cognitive change	C	The cognitive deficits do not occur exclusively in the context of a delirium	Informant reports deficits started 6 months or more ago, *or* informant reported delirium signs present for less than duration of cognitive change
D	The cognitive deficits are not better explained by another mental disorder	PHQ-9 < 10 *and* no informant-reported history of schizophrenia or other psychosis	D	The cognitive deficits are not better explained by another mental disorder	PHQ-9 < 10 *and* no informant-reported history of schizophrenia or other psychosis

^a^Informant questions of everyday cognitive difficulties and worsening modeled on DSM-5 ‘Examples of symptoms or observations’ for neurocognitive domains

^b^Praxis: ideomotor praxis *z* score ≤ –2.0; gnosis: BNT-15 *z* score ≤ –2.0, and COWAT > –2.0

^c^HRS-IADL are self-report questions [40]

^d^Informant endorses any of Dysexecutive Questionnaire (DEX-Q) items 9, 11, 13 or 20 with frequency of ‘sometimes’ or more, *or* endorses any of the following: “Behave out of character or inappropriately”; “Unconcerned or unaware of how others feel”; “Less participation in social functions for reasons other than physical”, “Lost special skills interests or hobbies”

^e^Informant endorses any of following: “Difficulty with familiar tasks like parking a car, assembling objects, sewing etc.” “Difficulty using familiar tools or equipment”, “Get lost in familiar places”

^f^Tests available for determining objective decline over time: CVLT immediate and delayed recall, Digits back, SDMT, Purdue Pegboard, COWAT and Trails B, simple and complex RT

*DSM* Diagnostic and Statistical Manual of Mental Disorders, *IADL* instrumental activities of daily living, *IQCODE* Informant Questionnaire of Cognitive Decline in the Elderly, *MAC-Q* Memory and Cognitive Questionnaire, *MCI* mild cognitive impairment, *PHQ* Patient Health Questionnaire

**Table 2:** Operationalization of DSM-5 mild NCD and IWG MCI within the algorithm

DSM-5 mild neurocognitive disorder			IWG MCI		
Criteria		Algorithm	Criteria		Algorithm
A. Both of the following:					
A1	Concern of self or informant of significant cognitive decline in one or more cognitive domains	MAC-Q >24 *or* IQCODE >3.31 *or* recent doctor’s consultation about cognitive change *or* informant reported worsening of everyday cognitive function^a^	1	Participant is not normal and not demented	Does not meet criteria for DSM-IV dementia or DSM-5 major NCD and all cognitive domains not in normal range (*z* score ≤ –1.0)
A2	Modest impairment in cognitive performance in one or more cognitive domains	Mean *z* score for one or more of the following domains is > –2.0 to ≤ –1.0: complex attention, executive function, learning and memory, language, perceptual/motor, social cognition, *or* mean decline in performance between waves 3 and 4 that is > –2.0 to ≤ –1.0 SD below norms on select tests^b^	2. Either or both of the following (2a *and*/o*r* 2b):		
			2a	Self and/or informant report of cognitive decline *and* Impairment on objective cognitive tasks	MAC-Q >24 *or* IQCODE >3.31 *or* recent doctor’s consultation about cognitive change or informant reported worsening in everyday cognition. *And* mean *z* score ≥ –2.0 and ≤ –1.0 for one or more of: memory, complex attention, executive function, language, and perceptual/motor
B	A1 and A2 do not interfere with capacity for independence in everyday life	No HRS-IADL^c^ problems due to memory, or self-reported need for household/personal care help, *or* Bayer IADL <3.12, *and* (if A2 social cognition impaired, then no informant-reported^d^ change in social behaviors or emotion recognition)	2b	Evidence of decline over time on objective cognitive tasks	Mean decline in performance between waves 3 and 4 that is > –2.0 to ≤ –1.0 SD below norms on select tests^b^
C	The cognitive deficits do not occur exclusively in the context of a delirium	Informant reports deficits started 6 months or more ago, *or* informant-reported delirium signs present for less than duration of cognitive change	3	Preserved basic activities of daily living *or* minimal impairment on complex IADLs	No difficulty with Bayer IADL items 2, 4, and 11 *or* no self-reported need for personal care help, *or* Bayer IADL <3.12
D	The cognitive deficits are not better explained by another mental disorder	PHQ-9 < 10 *and* no informant-reported history of schizophrenia or other psychosis			

^a^Praxis: ideomotor praxis *z* score ≤ –2.0; gnosis: BNT-15 *z* score ≤ –2.0, and COWAT > –2.0

^b^Tests available for determining objective decline over time: CVLT immediate and delayed recall, Digits back, SDMT, Purdue Pegboard, COWAT and Trails B, simple and complex RT

^c^HRS-IADL are self-report questions [40]

^d^Informant endorses any of DEX-Q items 9, 11, 13 or 20 with frequency of ‘sometimes’ or more, *or* endorses any of the following: “Behave out of character or inappropriately”; “Unconcerned or unaware of how others feel”; “Less participation in social functions for reasons other than physical”, “Lost special skills interests or hobbies”

*DSM* Diagnostic and Statistical Manual of Mental Disorders, *IADL* instrumental activities of daily living, *IQCODE* Informant Questionnaire of Cognitive Decline in the Elderly, *IWG* International Working Group, *MAC-Q* Memory and Cognitive Questionnaire, *MCI* mild cognitive impairment, *NCD* neurocognitive disorder, *PHQ* Patient Health Questionnaire

### Expert diagnosis and consensus

Case files (*n* = 368) were reviewed by an experienced research neurologist (CM); these included neuropsychological test data, informant data, structural brain magnetic resonance imaging (MRI) scans to aid differential diagnosis of dementia subtypes (*n* = 54), a self-reported medication list, and contact details of the participant for further clarification of details relevant to diagnosis (*n* = 21). The neurologist based her decisions on all available data, guided by the DSM-5 NCD, DSM-IV, and MCI diagnostic criteria, and used clinical judgement to determine whether each criterion was supported by the data. Inter-rater reliability with an experienced psychiatrist (RK) independently reviewing a subsample of 29 cases indicated high agreement for dementia (DSM-IV and DSM5 major NCD: κ = 0.79, 95% confidence interval (CI) 0.54–1.0, *p* < 0.01), and moderate agreement for mild cognitive disorders (MCI and DSM5 mild NCD: κ = 0.47, 95% CI 0.13–0.73, *p* < 0.01) which are within the ranges reported in field trials [7, 11, 38].

Further to estimating inter-rater reliability, consensus diagnosis was conducted by the two physicians and a neuropsychologist (RE) on complex cases identified as meeting at least one of the following criteria: (1) comorbid depression (moderate to severe on PHQ-9); (2) other comorbid psychiatric conditions; (3) stroke; (4) dementia or DSM-5 major NCD without memory impairment. A total of *n* = 60 met the above criteria and diagnoses were reviewed for consensus.

### Statistical analysis

To evaluate the accuracy of algorithmic classification relative to the expert diagnoses, we used the binary algorithmic criteria (equally weighted) as predictors of expert diagnosis in logistic regression models, saving the model predicted probabilities. We then conducted receiver operating characteristic (ROC) analyses of each probability variable against the corresponding binary diagnosis variable. Cross-tabulation and kappa (κ) statistics were used to evaluate agreement between algorithmic and expert diagnosis, with bootstrapping of 1000 samples to estimate 95% CIs on the kappa. Overlap between the different diagnostic criteria when used by clinicians was examined using crosstabs. Generalized linear models (GLM) were used to examine mean differences in each cognitive domain between diagnostic groups identified by the clinicians.

## Results

### Participant demographics

Compared to those selected as ‘normal’ at screen 1 (*n* = 1021) and the algorithmic screen (*n* = 255), the sample selected for expert review (*n* = 368) had significantly lower MMSE scores (27.4 (standard deviation (SD) = 2.7) vs 29.2 (SD = 0.95), *p* < 0.001), greater depressive symptomatology on PHQ-9 (3.8 (SD = 3.9) vs 2.7 (SD = 3.1), *p* < 0.001), higher frequency of males (56.8% vs 50.5%, *p <* 0.05), and similar frequency of carrying at least one *APOE e4* allele (31% vs 26%, *p* = 0.052). There were no differences in age (75.2 years (SD = 1.6) vs 75.1 (SD = 1.5), *p >* 0.10) or dementia family history (23% vs 22%, *p* > 0.10).

### Accuracy of psychometric algorithm for DSM-5 NCDs, DSM-IV dementia, and MCI

**Fig. 2:**
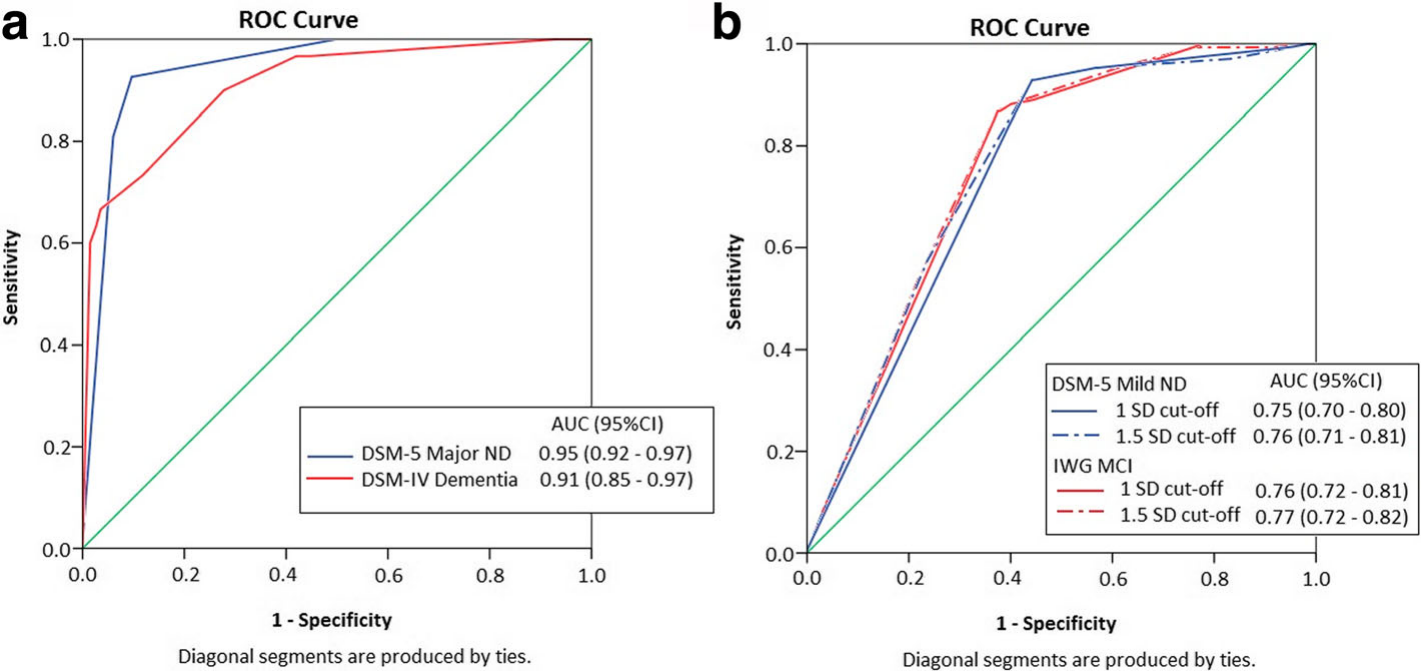
Receiver operating curve (*ROC*) for discriminating clinically diagnosed categories from algorithm-based categories (*n* = 368). **a** Dementia and major neurocognitive disorder (*ND*). **b** International Working Group mild cognitive impairment (*IWG MCI*) and mild ND with comparison between cognitive cut-offs (1.5 SD and 1.0 SD). *AUC* area under the curve, *CI* confidence interval, *DSM* Diagnostic and Statistical Manual of Mental Disorders

The algorithm classified 72 cases as meeting criteria for DSM-5 major NCD. ROC analysis of logistic regression-derived algorithmic probability of diagnosis against expert diagnosis indicated excellent accuracy (area under the curve (AUC) = 0.95, 95% CI 0.92–0.97) (Fig. 2a). Of these 72 cases, 54 (75%) were confirmed by the clinicians, representing an overall high level of agreement (κ = 0.72, 95% CI 0.62–0.80).

Twenty-seven cases were diagnosed as DSM-IV dementia by the algorithm. ROC analysis indicated excellent accuracy relative to expert-diagnosed DSM-IV dementia (AUC = 0.91, 95% CI 0.85–0.97) (Fig. 2a). Of the 27 cases, 19 (70.4%) were expert-confirmed, yielding a high level of agreement (κ = 0.64, 95% CI 0.47–0.78).

When a cut-off of 1 SD was applied to identify cognitive impairment in the mild range, the algorithm classified 220 cases as DSM-5 mild NCD, of which 141 (64.1%) were expert-confirmed (κ = 0.43, 95% CI 0.33–0.52). ROC analysis revealed very good prediction of expert diagnosis (AUC = 0.75, 95% CI 0.70–0.80) (Fig. 2). When a cut-off of 1.5 SD was applied, 143 cases were classified as DSM-5 mild NCD, of which 96 (67.1%) were expert-confirmed (κ =0.34, 95% CI 0.22–0.43). ROC analysis showed good prediction of expert diagnosis (AUC = 0.76, 95% CI 0.71–0.81) (Fig. 2b).

For MCI, algorithmic diagnosis using a cut-off of 1 SD resulted in 190 cases being classified with 113 (59.5%) confirmed by expert diagnosis (κ =0.42, 95% CI 0.33–0.51). ROC analysis indicated very good accuracy (AUC = 0.76, 95% CI 0.72–0.81) (Fig. 2b). When a cut-off of 1.5 SD was applied, 124 cases were identified, with 76 (61.3%) being expert confirmed (κ = 0.32, 95% CI 0.22–0.41), with ROC indicating very good prediction (AUC = 0.77, 95% CI 0.72–0.82) (Fig. 2b).

### Predictive value of individual algorithmic criteria for identifying algorithm and expert diagnosis

Positive (PPV) and negative predictive values (NPV) of individual criteria (see Additional file 1: Table S2) are presented as functions of source of diagnosis (i.e., algorithm or expert). Predictive values were obtained using crosstabs of observed frequencies of those meeting each criterion against those achieving diagnosis. In general, the pattern of PPV for individual criteria was similar for algorithmic and expert diagnosis.

### Sensitivity analysis

Informant data was unavailable for 59 (9.5%) of the cases selected by screen 1 (Fig. 1). Within this group, the distribution of dementia/major NCD (*n* = 3 (5.1%)) or MCI/mild NCD (*n* = 12 (20.3%)) was similar to that in the full sample (*n* = 71 (4.3%) and *n* = 196 (11.9%), respectively) (χ^2^(2) = 3.96, *p* = 0.14). To examine the impact of missing data on the analyses of algorithm accuracy, cross-tabulation and κ statistics were obtained for only those that had informant data (*n* = 346). Agreement was similar to that found in the full sample: major NCD κ = 0.73, 95% CI 0.63–0.82; mild NCD κ = 0.43, 95% CI 0.33–0.51; dementia κ = 0.63, 95% CI 0.44–0.78; and MCI κ = 0.43, 95% CI 0.34–0.53.

### Overlap between expert diagnosed DSM-5 NCDs and DSM-IV dementia and MCI

Cross-tabulation of expert-diagnosed DSM-5 major NCD against DSM-IV dementia showed a moderate level of overlap (κ =0.49, standard error (SE) = 0.06, *p* < 0.001) (Table 3). Of the 30 cases meeting criteria for DSM-IV dementia, 27 (90%) also met criteria for DSM-5 major NCD. The three cases meeting DSM-IV dementia but not DSM-5 major NCD both received AD etiological specifiers and met criteria for DSM-5 mild NCD. The DSM-5 identified 41 additional cases as dementia, representing a 127% increase in dementia diagnosis in the sample relative to DSM-IV, and a high positive predictive value (PPV = 0.88; NPV = 0.90). These additional cases included a few with vascular, fronto-temporal, and Parkinson’s specifiers. They also had a higher rate of previous diagnoses (36.6%) relative to cases without any expert-diagnosed dementia (3.4%) (*p* < 0.001), and a similar rate to those meeting criteria for both DSM-5 and DSM-IV dementia diagnoses (40%) (*p* > 0.05). Cases qualifying for both DSM-5 major NCD and DSM-IV dementia were also more likely to carry at least one *APOE e4* allele (55.2%) compared to those meeting only the DSM-5 major NCD diagnosis (14.6%) (*p* < 0.001), with the latter being statistically not different from the *APOE e4* allele frequency in cognitively normal participants (25.8%) (*p* > 0.05).

**Table 3:** Overlap between expert diagnoses using DSM-5 criteria and DSM-IV for dementia and MCI

DSM-IV dementia	DSM-5 major NCD		MCI	DSM-5 mild NCD	
	No	Yes		No	Yes
No	297 (87.9%)	41 (12.1%)	No	172 (76.8%)	52 (23.2%)
Yes	3 (10.0%)	27 (90.0%)	Yes	25 (17.4%)	119 (82.6%)
Kappa	0.494 (0.063)	*p* < 0.001	Kappa	0.575 (0.043)	*p* < 0.001
Specifiers	DSM-5	DSM-IV	Specifiers/subtypes	DSM-5	MCI
Probable Alzheimer’s	17 (25%)	18 (60%)	Amnestic—single	42 (24.6%)	46 (31.9%)
Possible Alzheimer’s	8 (11.8%)	7 (23.3%)	Amnestic—multiple	40 (23.4%)	49 (34.0%)
Probable vascular	3 (4.4%)	1 (3.3%)	Non-amnestic—single	27 (15.8%)	36 (25%)
Possible vascular	4 (5.9%)	0 (0%)	Non-amnestic—multiple	9 (5.3%)	12 (8.3%)
Parkinson’s	4 (5.9%)	3 (10%)	Probable Alzheimer’s	2 (1.2%)	0 (0%)
Lewy body	1 (1.5%)	1 (3.3%)	Possible Alzheimer’s	2 (1.2%)	0 (0%)
Fronto-temporal	3 (4.4%)	0 (0%)			
Unspecified	28 (41.2%)	0 (0%)	Unspecified	49 (34.4%)	–
Total	68	30	Total	171	144

*DSM* Diagnostic and Statistical Manual of Mental Disorders, *MCI* mild cognitive impairment, *NCD* neurocognitive disorder

There was a moderate level of overlap (κ = 0.58, SE = 0.04) between DSM-5 mild NCD and MCI diagnosis. Of the 144 cases qualifying for MCI, 119 (82.6%) were also given DSM-5 mild NCD diagnosis. The 25 MCI cases missed by DSM-5 mild NCD did not qualify for a diagnosis of DSM-5 major NCD or any other diagnostic category. They were mostly of the amnestic multi-domain (*n* = 9) and non-amnestic single domain (*n* = 9) subtypes. An additional 52 cases also received mild NCD diagnosis, representing an overall 19% increase in mild cognitive disorder diagnoses in our sample (PPV = 0.78; NPV = 0.82).

### Characterization of neuropsychological profiles as a function of expert diagnosis overlap

**Fig. 3:**
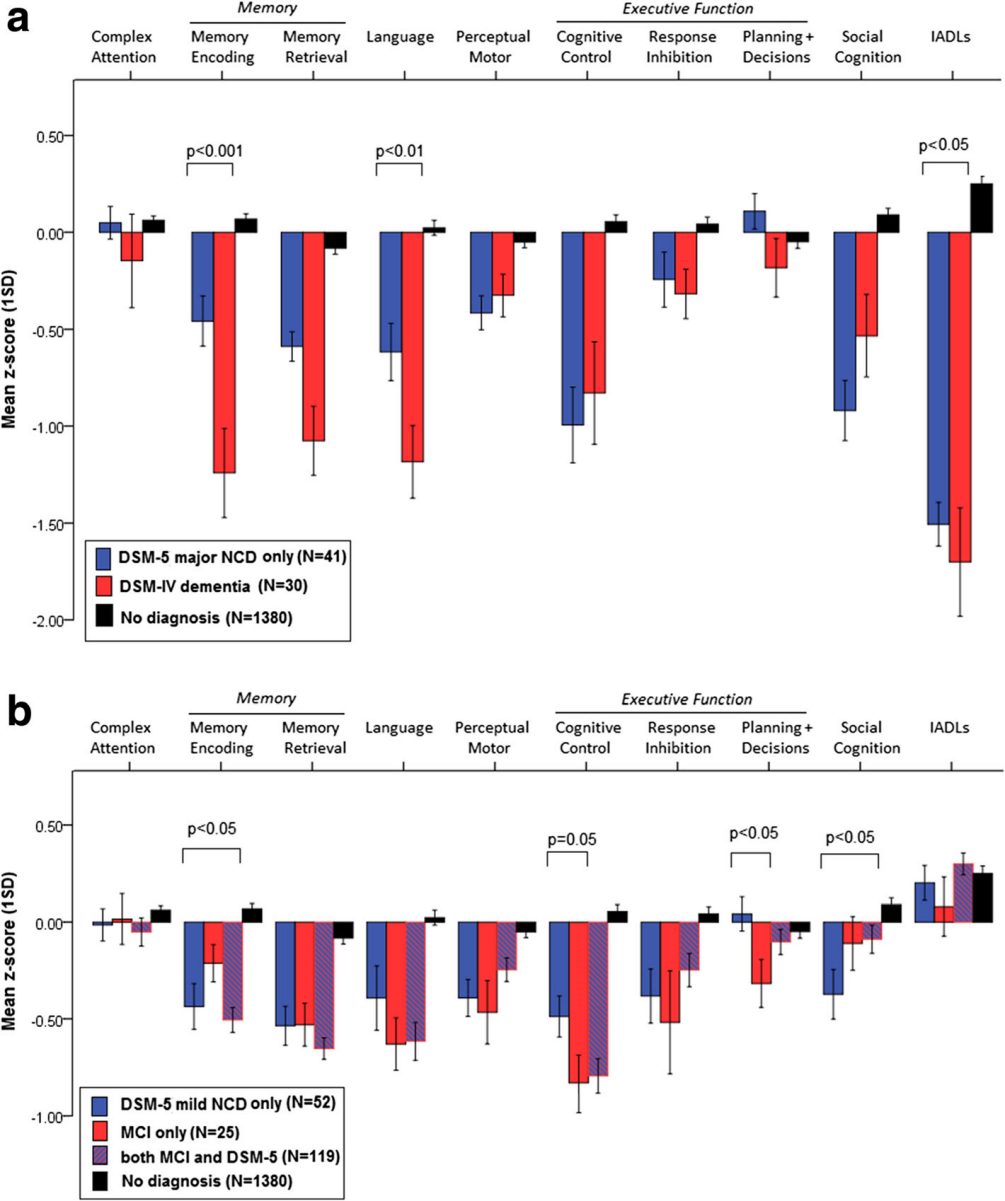
Cognitive profiles as a function of diagnostic category. **a** Mean *z* score (standardized relative to education- and gender-stratified norms for the whole PATH sample) for tests in each cognitive domain as a function of diagnostic category: DSM-IV dementia (*n* = 30), DSM-5 major NCD only (*n* = 41), no diagnosis (*n* = 1380). **b** Mean *z* score for cognitive domain as a function of diagnosis: MCI only (*n* = 25), DSM-5 mild NCD only (*n* = 52), both MCI and DSM-5 mild NCD (*n* = 119). Error bars represent 1 standard error (*SD*). *Cognitive Control* Trails B and Digits Backward; *Response Inhibition* Stroop, Go NoGo test; *Planning and Decision Making* Zoo Map sequencing and error, Dice test safe choices and strategy changes; *Memory Encoding* California Verbal Learning Test Delayed Recall, Recognition Hits and Misses; *Memory Retrieval* California Verbal Learning Test Trial 1, Trial 3 and Delayed Recall. *DSM* Diagnostic and Statistical Manual of Mental Disorders, *IADLs* instrumental activities of daily living, *MCI* mild cognitive impairment, *NCD* neurocognitive disorder

A series of GLMs compared neurocognitive profile as a function of diagnosis. GLM analysis revealed that cases diagnosed with only DSM-5 major NCD had significantly better language (*p* < 0.01), memory encoding (*p* < 0.001), and IADL function (*p* < 0.05) compared to cases that also met DSM-IV dementia criteria (Fig. 3a).

Figure 3b presents neuropsychological profiles as a function of DSM-5 mild NCD and MCI. Relative to normal controls, cases with either DSM-5 mild NCD, MCI, or both performed poorly in all domains except IADLs. Relative to cases with only MCI, cases given only DSM-5 mild NCD diagnoses had poorer memory encoding (*p* < 0.05) and poorer social cognition (*p* < 0.05), but better planning and decision making (*p* < 0.05).

## Discussion

### Algorithm accuracy

We report the first algorithmic approach to classifying DSM-5 NCDs. The algorithm used had good accuracy when classifying major NCD (κ = 0.72, AUC = 0.95) and DSM-IV dementia (κ = 0.64, AUC = 0.91) and was reasonably accurate when classifying MCI (κ = 0.42, AUC = 0.75) and mild NCD (κ = 0.43, AUC = 0.76). The findings indicate that a psychometric algorithm is capable of predicting clinical diagnosis in a population-based sample of older adults, and is consistent with previous work suggesting better algorithmic prediction of more severe diagnoses compared to milder diagnoses [5, 7]. Our findings also support field trials of the DSM-5 NCD [11] which found that the reliability of mild NCD was generally lower and less consistent than that of major NCD, which was very good. The algorithm for DSM-5 criteria produced slightly more accurate prediction of expert diagnosis compared to DSM-IV dementia criteria or IWG MCI criteria, supporting our hypothesis that the clearer, more structured DSM-5 criteria may be easier to operationalize. Agreement between algorithmic and expert diagnosis ranged between κ = 0.42 and κ = 0.72, consistent with previously published algorithms [5, 7, 8]. We also found that the cognitive cut-off used to define mild impairment (either 1.0 or 1.5 SD) had minimal impact on the rate of diagnosis of either DSM-5 mild NCD or IWG MCI diagnosis.

The individual diagnostic criteria that were predictive of expert-diagnosed major NCD and DSM-IV dementia were similarly predictive of algorithm-defined major NCD and dementia, with cognitive impairment and IADL impact having the highest PPV. Individual criteria were less predictive for the mild diagnoses, but those with highest PPVs included cognitive impairment, subjective concern, and exclusion of dementia (in the case of MCI). The lower predictive value of algorithmic criteria for delirium and other disorders for expert diagnoses suggest greater reliance on clinical judgement when determining their likely impact.

### DSM-5 overlap with DSM-IV and MCI, and comparison of neurocognitive profiles

We also found that expert diagnosis of dementia according to DSM-5 had excellent overlap with DSM-IV (90%); however, a large number of additional cases were identified by DSM-5 resulting in a 127% increase in diagnosis. This confirms the findings of Tay et al. [14] in a memory clinic sample (*n* = 234) where they found that DSM-5 major NCD criteria captured all cases of DSM-IV dementia, but with an additional 39.7% cases. These additional cases, however, had a similar rate of previous diagnoses (either MCI or dementia) to cases meeting only DSM-IV dementia, and a significantly higher rate than those without dementia, suggesting the more inclusive criteria captured additional cases with similarly chronic deficits.

Aside from the different populations, our higher rate of additional diagnosis may reflect our use of more detailed neurocognitive measurement, detailed informant report, and inclusion of etiological specifiers and structural MRI evidence. In the absence of sufficient data on the degree of impairment or biological evidence of change, cases not meeting DSM-IV dementia are more likely to be labeled as mild. While Tay et al. [14] labeled as MCI most of those who were DSM-5 major NCD but not DSM-IV dementia, none of our additional DSM-5 major NCD cases met criteria for MCI. Instead, they were more likely to receive a vascular specifier, fronto-temporal or Parkinson’s dementia. Although memory impairment was less severe for the group with only DSM-5 major NCD, the relative severity of impairment in other cognitive domains, as well as reported impact on IADLs, show that this group should be considered as dementia. Thus, our findings suggest that additional dementia cases identified by DSM-5 are not necessarily at a milder stage but present with a different neuropsychological profile, and possibly different etiologies, compared to cases meeting dementia criteria for both DSM-5 and DSM-IV where the pattern of impairment and *APOE e4* allele distribution is more supportive of AD. Future research including additional biomarkers will enable evaluation of this finding.

Although the mild NCD criteria were not developed as an explicit replacement for IWG MCI, in the context of ageing-associated progressive NCDs, clinicians may consider them as an alternative. Accordingly, diagnosis of DSM-5 mild NCD was highly sensitive to MCI (83%) and showed a moderate agreement with MCI diagnosis (κ = 0.58), albeit with an overall 19% increase in the rate of diagnosis. This contrasts with Tay et al. [14] who reported a decrease of 54% using DSM-5 mild NCD criteria, and attributed this to difficulties defining the level of IADL impairment appropriate for mild NCD. Population-based samples are more likely to contain individuals with very little functional impairment but sufficient cognitive deficits and decline to warrant a mild NCD diagnosis.

Luck et al. [15] reported a much higher agreement between MCI and DSM-5 mild NCD, but assessed each neurocognitive domain with a single test. Our use of a range of tests and obtaining average performance across the domain is likely more sensitive to true impairment but more variable. In fact, in our sample, 17.4% of MCI cases failed to be captured by DSM-5, and there were differences in neuropsychological profile, such that cases meeting only DSM-5 mild criteria had poorer social cognition and memory, supporting previous findings [15], but better performance on planning and decision-making. This suggests the inclusion of a greater range of neurocognitive domains in DSM-5, and particularly the inclusion of social cognition as a criterion, may help capture impaired individuals not detected by MCI criteria. Follow-up studies are required to examine the progression and predictive value of these cases.

Our study is limited by expert diagnosis based on case file review rather than clinical interview; however, this meant that our clinical diagnoses were based on the same data as those operationalized in the algorithm. Nevertheless, further work is required to validate these findings in independent data sets. Strengths include the large, population-based sample, detailed neurocognitive assessment, comparison of different cognitive cut-offs, and a systematic approach to collecting and analyzing evidence for impairment. The findings suggest that clinicians, trialists, and epidemiologists using the DSM-5 criteria should expect higher estimates of disease prevalence and incidence, and the ability to capture a broader range of etiologies and severities compared to DSM-IV and MCI. The findings also suggest that while MCI and mild NCD do overlap, MCI is not fully captured within the mild NCD construct. A similar pattern may be apparent for the forthcoming ICD-11 criteria if it adopts an approach analogous to DSM-5 [39].

## Conclusions

In summary, an algorithm-based approach to DSM-5 diagnosis of NCD is feasible in cohort studies. This approach is more accurate at identifying major NCD than mild NCD. DSM-5 is more inclusive of the variety of clinical profiles of major NCD, resulting in higher rates of diagnosis but with good negative predictive power. The findings have implications for understanding the impact on rates of diagnosis when using the revised diagnoses.

## Additional file

Additional file 1: Supplementary methods detailing neuropsychological test battery, criteria for screen 1, and Tables S1 and S2. (DOCX 26 kb)

## Abbreviations

AD: Alzheimer’s Disease
AUC: Area under the Curve
CI: Confidence interval
DEX-Q: Dysexecutive Questionnaire
DSM-5/-IV/-III-R: Diagnostic and Statistical Manual of Mental Disorders, 5th Edition/4th Edition/3rd Edition Revised
GML: Generalized linear models
IADL: Instrumental activities of daily living
IQCODE: Informant Questionnaire of Cognitive Decline in the Elderly
IWG: International Working Group
MAC-Q: Memory and Cognitive Questionnaire
MCI: Mild cognitive impairment
MMSE: Mini-Mental Status Examination
MRI: Magnetic resonance imaging
NCD: Neurocognitive disorder
NPI: Neuropsychiatric Inventory
NPV: Negative predictive values
PHQ: Patient Health Questionnaire
PPV: Positive predictive values
ROC: Receiver operating characteristic
SD: Standard devation
SE: Standard error
