# Supplementary material for: Evaluation of a research diagnostic algorithm for DSM-5 neurocognitive disorders in a population-based cohort of older adults
Source: Alzheimers Res Ther. 2017 Mar 4;9:15. doi: 10.1186/s13195-017-0246-x (PMC5336665; doi:10.1186/s13195-017-0246-x)
Supplement: Additional file 1: — Supplementary methods detailing neuropsychological test battery, criteria for screen 1, and Tables S1 and S2. (DOCX 26 kb) [file 13195_2017_246_MOESM1_ESM.docx]

**Supplementary Methods**

***Neurocognitive Measures and Domains***

Complex Attention: *Symbol Digits Modalities Test* (19) oral version measured processing speed and selective attention. *Trail Making Test A* from the Halstead-Reitan Battery (20) measured processing speed and attention. *Reaction Time Test* (21) measured participants’ mean reaction time to depress a button in response to a light (Simple RT) and to choose and depress one of two buttons in response to the location of a light (Choice RT).

Executive Function: *Digit Span Backwards* from the Wechsler Memory Scale (22), assessed verbal working memory. *Trail Making Test B* assessed mental flexibility and executive control of attention (TMT B). *Stroop Colour Word Test* – Victoria Version (23) measured response inhibition. *Zoo Map* from the Behavioral Assessment of the Dysexecutive Syndrome (BADS) battery (24) assessed planning. *Game of Dice Test* (25) assessed decision-making under explicit risk conditions. Learning and Memory: *California Verbal Learning Test* (26) Three learning trials, a delayed recall trial and delayed recognition were used. *Benton Visual Retention Test* (27) (Administration B) measured immediate visual memory. Language: *Letter Fluency* from the Halstead-Reitan Battery (20) assessed verbal fluency. *Boston Naming Test-15 item* (28) measured confrontation naming. *Spot The Word Test* (29) was used to measure verbal knowledge. Perceptual Motor: *Purdue Pegboard* (30) assessed psychomotor speed and dexterity. *Ideomotor Apraxia Test (IAT)* (31) assessed the ability to imitate gestures and pantomime the use of objects. *Benton Visual Retention Test* (27) (Administration C) assessed visuo-construction. Social Cognition: *Reading the Mind in the Eyes* (32) was used to assess Theory of Mind and ability to identify emotion and mental state from faces.

***Criteria for Screen 1***

**A)** previous PATH diagnosis of cognitive disorder (including dementia) at Wave 1, 2 or 3;

OR

**B)** either:

i) evidence of cognitive impairment: Mini-Mental State Exam (MMSE) ≤ 24 at Wave 4; or

ii) performance on one or more cognitive tests ≤ 6.7th percentile at Wave 4 (Immediate recall, Delayed recall, SDMT, F words, A words, Boston Naming Test, Simple RT, Choice RT, Pegboard dominant, Pegboard non-dominant, Pegboard both, Digits Back, Trails B, Stroop Words, Stroop Color-Word);

AND either:

iii) subjective decline: scores ≥ 25 on the (MAC-Q); or

iv) evidence of decline: >3 point decline in MMSE score since Wave 3, or

v) evidence of consistent cognitive impairment across time: MMSE ≤ 24 at Waves 3 and 4.

Table S1. Assessment of neurocognitive domains

| Domain | Objective Measures | Example questions from Informant Interview |
| --- | --- | --- |
| Complex Attention | Symbol Digit Modalities | Slower to complete normal activities, difficulty thinking with background noise or activity. |
|  | Trail Making Test A |  |
|  | Simple Reaction Time |  |
|  | Choice Reaction Time |  |
| Executive Function | Digit Span Backwards | Dysexecutive Symptoms Questionnaire (BADS) |
|  | Trail Making Test B |  |
|  | Stroop Color Word Test |  |
|  | Zoo Map (BADS) |  |
|  | Game of Dice Test |  |
|  | Go No-Go test |  |
| Learning and Memory | California Verbal Learning Test | Difficulty recalling recent events, following a movie/book, learning new things. Forget to turn off stove, repeat themselves in conversation, loses things, difficulty remembering faces or names. Greater reliance on memory aids. |
|  | Benton Visual Retention Test |  |
|  |  |  |
| Language | Letter Fluency | Trouble findings words, difficult to understand what they are talking about. |
|  | Boston Naming Test (BNT-15) |  |
|  | Spot the Word Test |  |
| Perceptual Motor | Purdue Pegboard Test | Difficulty using familiar tools, parking a car, sewing or building etc. Reliance on maps to navigate. Gets lost. |
|  | Benton Visual Retention Copy |  |
|  | Ideomotor Praxis |  |
| Social Cognition | Reading the Mind in the Eyes | Behave inappropriately, less participation in social activities, unconcerned or unaware of others feelings. Social DEX-Q items. |

Table S2. Predictive value of algorithmic diagnostic criteria for detecting diagnosis based on Algorithm (n=1495), and based on clinical diagnosis (n=368).

|  | **Algorithmic diagnosis** | | **Expert diagnosis** | |  | **Algorithmic diagnosis** | | **Expert diagnosis** | |
| --- | --- | --- | --- | --- | --- | --- | --- | --- | --- |
|  | **PPV** | **NPV** | **PPV** | **NPV** |  | **PPV** | **NPV** | **PPV** | **NPV** |
| **DSM-5 major NCD** |  |  |  |  | **DSM-5 mild NCD** |  |  |  |  |
| A1. Subjective concern | 0.59 | 1.00 | 0.52 | 1.00 | A1. Subjective concern | 0.61 | 1.00 | 0.53 | 0.85 |
| **A2. Impairment (<2SD)** | **0.86** | **1.00** | **0.64** | **0.81** | **A2. Impairment (>-2 to < -1SD)** | **0.86** | **1.00** | **0.54** | **0.66** |
| **B. IADL impact** | **0.95** | **1.00** | **0.91** | **0.92** | **B. Min. IADL impact** | **0.54** | **1.00** | **0.63** | **0.90** |
| C. Not delirium | 0.50 | 1.00 | 0.50 | 1.00 | C. Not delirium | 0.50 | 1.00 | 0.50 | 1.00 |
| D. Exclude other disorder | 0.50 | 1.00 | 0.50 | 0.67 | D. Exclude other disorder | 0.50 | 1.00 | 0.55 | 0.72 |
| **DSM-IV Dementia** |  |  |  |  | **MCI** |  |  |  |  |
| **A1. Memory impaired** | **0.90** | **1.00** | **0.83** | **0.74** | **A. Not normal/dement** | **0.96** | **1.00** | **0.69** | **0.84** |
| A2. Other deficits (< -2SD) | 0.81 | 1.00 | 0.75 | 0.87 | B1. Report of decline | 0.53 | 0.63 | 0.51 | 0.72 |
| **B1. Functional impairment** | **0.91** | **1.00** | **0.81** | **0.80** | **B2. Impairment (>-2 to < -1SD)** | **0.72** | **0.81** | **0.62** | **0.77** |
| B2. Functional decline | 0.59 | 1.00 | 0.52 | 1.00 | B3. Obj. decline (>-2 to < -1SD) | 0.68 | 0.82 | 0.50 | 0.66 |
| C. Not delirium | 0.50 | 1.00 | 0.50 | 1.00 | C. Preserved ADLs | 0.49 | 0.48 | 0.55 | 0.59 |
| D. Not Axis 1 disorder | 0.50 | 1.00 | 0.50 | 1.00 | D. Min. IADL impact | 0.52 | 0.61 | 0.60 | 0.79 |

PPV: positive predictive value; NPV: negative predictive value. Bold items indicate top two most predictive/discriminative criteria for each diagnosis.
